# Immune Response of the Host and Vaccine Development

**DOI:** 10.3390/pathogens12050637

**Published:** 2023-04-24

**Authors:** Ewa Długosz, Agnieszka Wesołowska

**Affiliations:** 1 Division of Parasitology and Invasive Diseases, Department of Preclinical Sciences, Institute of Veterinary Medicine, Warsaw University of Life Sciences (SGGW), 02-787 Warsaw, Poland; 2 Museum and Institute of Zoology, Polish Academy of Sciences, Wilcza 64, 00-679 Warsaw, Poland

Vaccines are one of the greatest achievements of modern medicine, offering an effective way to fight and control infectious diseases. According to the World Health Organisation (WHO), up to 2–3 million lives are saved each year by the currently implemented immunisation programmes [1]. Still, there are many infectious diseases for which vaccines are not available. Whereas the first vaccines were based on attenuated or killed pathogens, purified pathogen antigens (subunit vaccines) were subsequently employed. Now, thanks to the tremendous advances in biotechnology, it is possible to use recombinant antigens or genetic vaccines for this purpose. The first step in vaccine development is the identification of a potential vaccine antigen. This can be achieved in many ways. Here, Bukhari et al. [2] provided the rationale behind the use of machine learning-based prediction methods in epitope selection. Epitope-based vaccines stimulate immune responses using B cell or T cell epitopes, and they have already demonstrated their tremendous potential in many studies.

The route of vaccine delivery is an important determinant of success. Vaccines can be administered in a number of ways, including intramuscular, subcutaneous, and intradermal routes. However, these routes do not mimic the natural entry of most pathogens, as many pathogens start infection through mucosal surfaces (e.g., respiratory or intestinal). The ideal vaccine against pathogens initiating infection through mucosa should induce effective host defenses at the site of pathogen entry. Therefore, an important goal is to efficiently stimulate mucosal immunity. Here, Karczmarzyk and Kęsik-Brodacka [3] describe strategies to obtain a mucosal vaccine against SARS-CoV-2 infection. Since new virus variants are still appearing, new vaccination strategies are highly awaited.

Ideally, vaccines should provide long lasting protection against the disease. However, for many vaccines, the effectiveness decreases over time. Here, Carr et al. [4] tested whether vaccinated mice retained resistance to HSV infection one year after the vaccine booster compared to a short-term efficacy study in which mice were challenged with HSV 30 days after booster vaccination. The study results highlight the need for long-term studies to estimate the duration of protective responses to infection. Developing a vaccine that provides proper long-term protection is a challenge.

As vaccines may carry some risk of adverse reactions, issues with vaccine safety are of major importance. They need to be evaluated carefully, and factors such as immunocompromised status, age, and comorbidities have to be considered. Here, Ramirez et al. [5] addressed the cardiac safety of mRNA vaccines against COVID-19 in patients with systemic lupus erythematosus and a previous history of myocarditis and reported that these vaccines can be safely administered.

One of the most successful human vaccines is a vaccine against smallpox. A global eradication programme launched by the WHO resulted in the eradication of smallpox in the human population. Still, other orthopoxviruses (OPXV) remain a threat. Repeated OPXV outbreaks have been reported worldwide, including monkeypox. As smallpox vaccine conferred cross-protection against other OPXV infections, the cessation of vaccination resulted in a waning immunity over time and may have consequences in the future. Those issues are reviewed extensively here by Gieryńska et al. [6].

In contrast to many anti-viral and anti-bacterial vaccines available for human and animal use, the paucity of anti-parasitic vaccines shows discrepancies in the development of effective immunisation strategies depending on the pathogen. Despite the tremendous efforts of scientists to create a vaccine that would successfully protect against parasitic diseases, only one is registered for humans (though there are and several for animals) [7,8]. The review presented in this Special Issue by Jerzak et al. [9] summarises the literature data on anti-Babesia vaccines and underlines the major problems which are faced by scientists. Among others, the lack of a reliable source of parasites for whole-parasite vaccine production, their genetic variation, which complicates the selection of antigens for subunit vaccines, and the scarcity of human-specific strong adjuvants complicate the process of vaccine development.

The identification of parasite-specific immunogenic molecules is one of the challenges faced by scientists aiming to produce subunit vaccines. One of the possible ways to reach this goal is the application of immunoproteomic methods. Here, Cybulska describes the identification of immunogenic proteins from *Trichinella britovi* using antibodies present in the meat juice of naturally infected carnivore hosts [10]. Using immunoblotting and liquid chromatography coupled with tandem mass spectrometry (LC-MS/MS), she identified over 200 proteins which induce the production of antibodies present in host tissues. These molecules could be considered as potential vaccine antigens, and their suitability for immunisation purposes can be evaluated in the future.

Despite all the data published to date, knowledge of the immune processes underlying host immunity to many pathogens is still insufficient. Therefore, several articles published in this Special Issue describe the complex mechanisms of host immune response regulation and the effect pathogen molecules play in the final outcome of the immune response.

First, Karabowicz et al. [11] review the role of Macrophage Inhibitory Factor (MIF) orthologs produced by parasitic nematodes in the process of host immune response modulation. Parasite MIFs mimic the activity of the corresponding host molecule and affect leukocyte migration, cytokine release, and macrophage polarisation. MIFs originating from two nematodes, *Teladorsagia circumcincta* and *Ancylostoma ceylanicum*, have also been used as vaccine antigens and have provided partial protection from infection in animal immunisation trials.

In another review, Bąska and Norbury [12] describe the role of Nuclear Factor Kappa B (NF-κB) transcription factor in the immune response against parasites. These pathogens have evolved numerous strategies to either up- or down-modulate NF-κB activity in order to facilitate their survival in the host. Many aspects of these mechanisms were described in detail with respect to parasite species and the type of host cells subjected to modulation.

Chrobak-Chmiel et al. [13] review the role of antimicrobial peptides and the effect of vitamin D3 in the regulation of innate immunity. Vitamin D3 induces the transcription of genes encoding pattern recognition receptors (PRRs) and activates cytokine production. These aspects are related to the ability of canine hosts with atopic skin disease to control secondary microbial infections. Although these issues are not strictly related to vaccines, the review gives some interesting insights into complex pathogen–host interactions.

In many cases, the immune response contributes to the pathology that develops during the infection process, and this should also be considered during vaccine development. Here, Zygner et al. [14] widely review the role of pro-inflammatory cytokines and chemokines in the development of anemia in dogs suffering from babesiosis. The authors describe several immune-related causes of anemia such as antibody production, erythrophagocytosis, oxidative damage of red blood cells, complement activation, and antibody-dependent cellular cytotoxicity, all of which are driven by pro-inflammatory cytokines and chemokines, especially IFN-γ, TNF-α, IL-6, and IL-8.

Although vaccination has a long history of success, there are still many infectious diseases for which vaccines are not yet available but are eagerly awaited. To fill this void, efforts to obtain the most effective strategies to prevent and control infectious diseases must be intensified. Any contribution in this area is highly anticipated. In particular, the identification of protective immune mechanisms, the selection of the best vaccine antigen candidates, and the identification of the optimal routes and regimens are important determinants of success. Hopefully, studies on host immune response will accelerate vaccine development and will result in new commercially available vaccines in the future.

## References

[1] World Health Organization (2020). Child Mortality and Causes of Death. WHO. https://www.who.int/gho/child_health/mortality/mortality_under_five_text/en/.

[2] Bukhari S.N.H., Jain A., Haq E., Mehbodniya A., Webber J. (2022). Machine Learning Techniques for the Prediction of B-Cell and T-Cell Epitopes as Potential Vaccine Targets with a Specific Focus on SARS-CoV-2 Pathogen: A Review. Pathogens.

[3] Karczmarzyk K., Kęsik-Brodacka M. (2022). Attacking the Intruder at the Gate: Prospects of Mucosal Anti SARS-CoV-2 Vaccines. Pathogens.

[4] Carr D.J.J., Berube A., Gershburg E. (2021). The Durability of Vaccine Efficacy against Ocular HSV-1 Infection Using ICP0 Mutants 0∆NLS and 0∆RING Is Lost over Time. Pathogens.

[5] Ramirez G.A., Batani V., Moroni L., De Luca G., Pizzetti G., Sala S., Peretto G., Campochiaro C., Della-Torre E., Bozzolo E.P. (2022). Cardiac Safety of mRNA-Based Vaccines in Patients with Systemic Lupus Erythematosus and Lupus-like Disorders with a History of Myocarditis. Pathogens.

[6] Gieryńska M., Szulc-Dąbrowska L., Struzik J., Gregorczyk-Zboroch K.P., Mielcarska M.B., Toka F.N., Schollenberger A., Biernacka Z. (2023). Orthopoxvirus Zoonoses—Do We Still Remember and Are Ready to Fight?. Pathogens.

[7] World Health Organization (2022). Malaria Vaccine: WHO Position Paper—March 2022. https://apps.who.int/iris/handle/10665/352337.

[8] Stutzer C., Richards S.A., Ferreira M., Baron S., Maritz-Olivier C. (2018). Metazoan Parasite Vaccines: Present Status and Future Prospects. Front. Cell. Infect. Microbiol..

[9] Jerzak M., Gandurski A., Tokaj M., Stachera W., Szuba M., Dybicz M. (2023). Advances in *Babesia* Vaccine Development: An Overview. Pathogens.

[10] Cybulska A. (2022). Immunoproteomic Analysis of *Trichinella britovi* Proteins Recognized by IgG Antibodies from Meat Juice of Carnivores Naturally Infected with *T. britovi*. Pathogens.

[11] Karabowicz J., Długosz E., Bąska P., Wiśniewski M. (2022). Nematode Orthologs of Macrophage Migration Inhibitory Factor (MIF) as Modulators of the Host Immune Response and Potential Therapeutic Targets. Pathogens.

[12] Bąska P., Norbury L.J. (2022). The Role of Nuclear Factor Kappa B (NF-κB) in the Immune Response against Parasites. Pathogens.

[13] Chrobak-Chmiel D., Golke A., Kwiecień E., Biegańska M.J., Dembele K., Dziekiewicz-Mrugasiewicz M., Czopowicz M., Kizerwetter-Świda M., Rzewuska M. (2023). Is Vitamin D3 a Worthy Supplement Protecting against Secondary Infections in Dogs with Atopic Dermatitis?. Pathogens.

[14] Zygner W., Gójska-Zygner O., Norbury L.J. (2023). Pathogenesis of Anemia in Canine Babesiosis: Possible Contribution of Pro-Inflammatory Cytokines and Chemokines—A Review. Pathogens.

